# Machine learning and radiomics for predicting efficacy of programmed cell death protein 1 inhibitor for small cell lung cancer: A multicenter cohort study

**DOI:** 10.1002/ctm2.1673

**Published:** 2024-06-05

**Authors:** Pulin Li, Ling Huang, Rui Han, Min Tang, Guanghe Fei, Daxiong Zeng, Ran Wang

**Affiliations:** ^1^ Department of Respiratory and Critical Care Medicine the First Affiliated Hospital of Anhui Medical University Hefei China; ^2^ Department of Infectious Disease Hefei Second People's Hospital Hefei China; ^3^ Department of Pulmonary and Critical Care Medicine Medical Center of Soochow University Dushu Lake Hospital Affiliated to Soochow University Suzhou China

Dear Editor,

Small cell lung cancer (SCLC) is notorious because of its rapid development, characterized by swift growth, high invasiveness, and limited treatment options.^1^, ^2^ Clinical trials have established the significant impact of integrating programmed cell death protein 1 (PD‐1) axis interdict with traditional platinum‐based chemotherapeutics for SCLC. This combination has been shown to enhance sustained overall survival, thereby setting a new standard for first‐line therapy in SCLC.^3^, ^4^ In 2022, Serplulimab, approved in China, is the sole PD‐1 inhibitor for extensive‐stage SCLC.^5^, ^6^ Therefore, our study aims to develop a novel model utilizing machine learning and radiomic techniques to predict the therapeutic response to PD‐1 inhibitors in SCLC patients.

Note that, 233 SCLC patients were recruited for this study from three medical centres (Center I: *n* = 107; Center II: *n* = 54; Center III: *n* = 72), the specific process is depicted in Figure S1. Based on the RECIST evaluation criteria,^7^ the objective response rate was used to assess response to PD‐1 inhibitors. The specific criteria are in Appendix 1. These SCLC patients were divided into the response cohort (*n* = 109) and the non‐response cohort (*n* = 124). Table S1 provides a detailed overview of the clinical characteristics of these patients. Multivariate logistic regression analysis uncovered a potential association between neuron‐specific enolase levels and the response of SCLC patients to PD‐1 inhibitors (odds ratio [OR]:.995, 95% confidence interval [95% CI]:.992–.999; *p* = .019), as detailed in Table S2 and illustrated in Figure S2.

From each region of interest analyzed using PyRadiomics,^8^ a total of 1,885 features were extracted. As described in Appendix 2, the feature selection process, which included a T‐test and Least Absolute Shrinkage and Selection Operator regression analysis, enabled us to identify the five most critical radiomic features for the construction of our predictive model. This process is depicted in Figure 1. Details regarding these selected radiomic features, along with relevant information, are presented in Figure S3 and Table S3.

**FIGURE 1:**
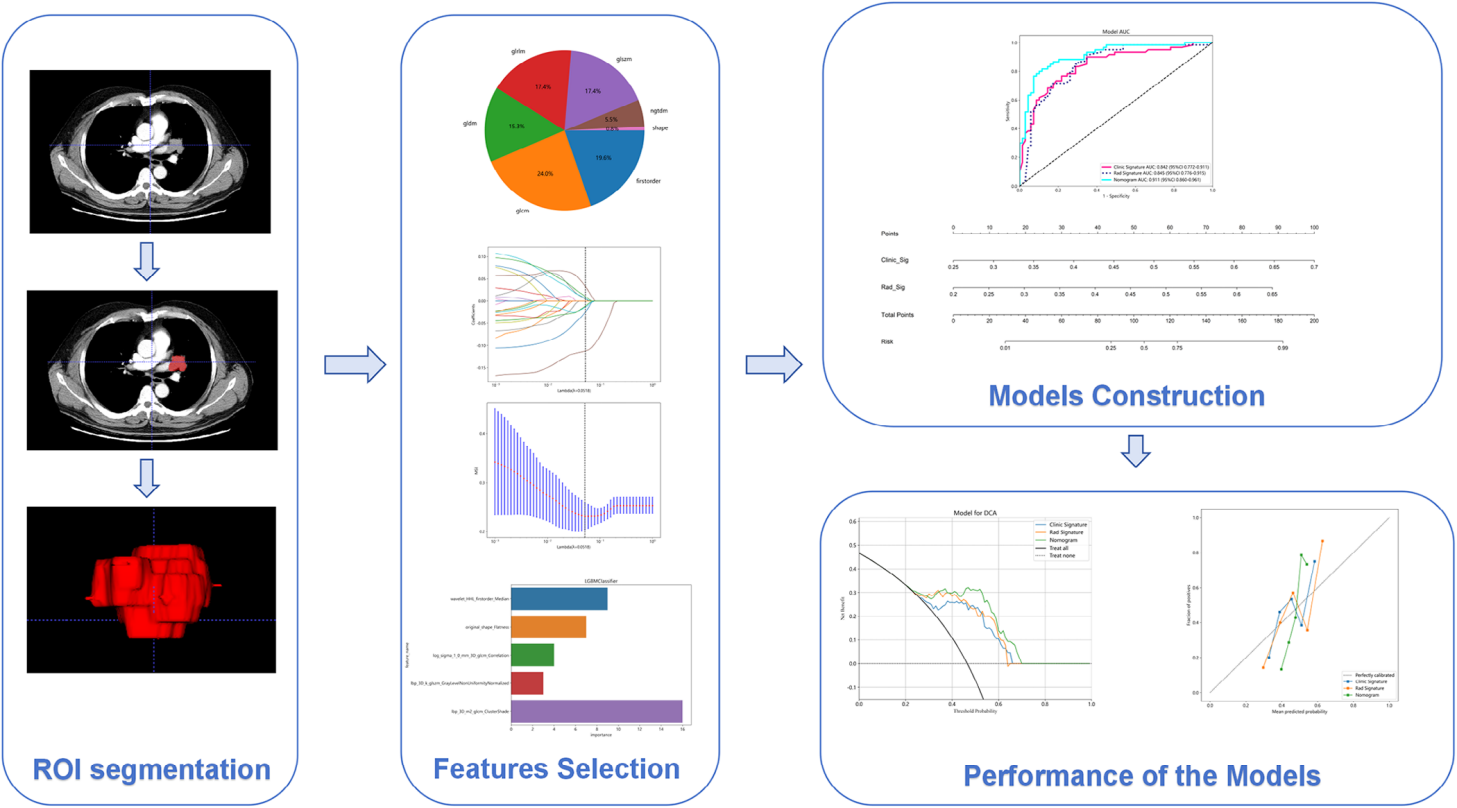
The workflow of this study.

Utilizing the previously identified radiomic features, we constructed a radiomics model within the training cohort employing the Light Gradient Boosting Machine method.^9^, ^10^ This model exhibited excellent predictive power, as evidenced by its performance in the training cohort, where it achieved an area under the curve (AUC) of.845 (95% CI = .776–.915). The model's robustness was further validated in two validation cohorts. In the internal validation cohort, the model achieved an AUC of.798 (95% CI = .637–.959), while in the external validation cohort, the AUC was.756 (95% CI = .644–.868). In contrast, the clinical model yielded varying AUC scores across the above three cohorts. Specifically, the AUCs were.842 (95% CI = .772–.911), 612 (95% CI = .403–.820) and.683 (95% CI = .559–.808).

To enhance predictive accuracy, we constructed radiomics clinical nomograms for the three cohorts, integrating both radiomics and clinical data. These nomograms demonstrated superior predictive value, with AUC scores of.911 (95% CI = .860–.961) in the training,.818 (95% CI = .665–.970) in the internal validation, and.798 (95% CI = .694–.902) in the external validation. The receiver operating characteristic curves and the radiomics clinical conjoint nomograms are illustrated in Figure 2 for a more comprehensive understanding of the models' performance.

**FIGURE 2:**
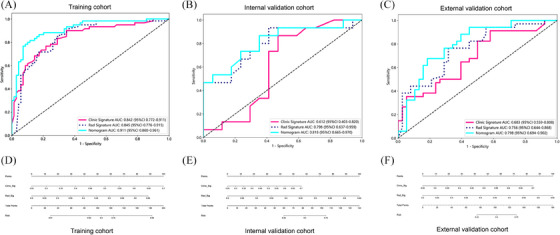
Receiver operating characteristic (ROC) curves analysis of the three models in the train and validation cohorts. (A) Training cohort, (B) internal validation cohort and (C) external validation cohort; Radiomics Clinical nomogram in the train and validation cohorts. (D) Training cohort, (E) internal validation cohort and (F) external validation cohort.

The models' performance was rigorously assessed through calibration curves and decision curve analysis (DCA). These methods provided insight into the models' predictive accuracy and clinical utility. The results from this assessment revealed that the radiomic model was consistently better than the clinical model in terms of predictive value across all three cohorts. This underscores the enhanced effectiveness of incorporating radiomic features into predictive modelling for SCLC patient response to PD‐1 inhibitors. Furthermore, the radiomics clinical nomograms, which integrate both radiomic and clinical data, demonstrated strong predictive capabilities in each of the three cohorts. This finding suggests their potential utility in guiding clinical decision‐making. The calibration and DCA curves, which provide a visual representation of these findings, are displayed in Figure 3. These graphs offer a clear depiction of the models' calibration and their net benefits across different threshold probabilities. In addition to predictive value, the radiomic model also showed superior accuracy and sensitivity compared to the clinical model. Table 1 presents a comprehensive summary of various performance metrics, including accuracy, sensitivity, precision, recall, and F1 scores, for all three models across the three cohorts. This tabulated data provides an in‐depth understanding of each model's performance and its implications for clinical application.

**FIGURE 3:**
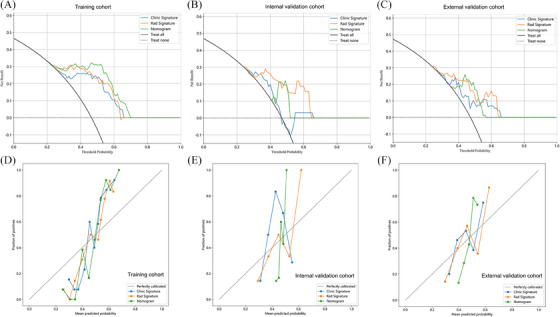
Decision curve analysis (DCA) curves of the three models in the train and validation cohorts. (A) Training cohort, (B) internal validation cohort and (C) external validation cohort; Calibration curves of the three models in the train and validation cohorts. (D) Training cohort, (E) internal validation cohort and (F) external validation cohort.

**TABLE 1:** Performance of the clinical model, radiomics model, and clinical‐radiomics nomogram in the training and validation cohorts.

Cohort	Accuracy	AUC	95% Cl	Sensitivity	Specificity	PPV	NPV	Precision	Recall	F1	Threshold	Model
Training cohort	0.767	0.842	0.772–0.911	0.652	0.900	0.882	0.692	0.882	0.652	0.750	0.554	Clinic model
	0.775	0.845	0.776–0.915	0.652	0.932	0.900	0.696	0.900	0.652	0.756	0.584	Rad model
	0.853	0.911	0.860–0.961	0.884	0.817	0.847	0.860	0.847	0.884	0.865	0.506	Nomogram
Internal validation cohort	0.688	0.612	0.403–0.820	0.529	0.867	0.818	0.619	0.818	0.529	0.643	0.624	Clinic model
	0.750	0.798	0.637–0.959	0.588	0.933	0.909	0.667	0.909	0.588	0.714	0.624	Rad model
	0.750	0.818	0.665–0.970	0.647	0.867	0.846	0.684	0.846	0.647	0.733	0.543	Nomogram
External validation cohort	0.653	0.683	0.559–0.808	0.421	0.912	0.842	0.585	0.842	0.421	0.561	0.623	Clinic model
	0.722	0.756	0.644–0.868	0.684	0.765	0.765	0.684	0.765	0.684	0.722	0.543	Rad model
	0.736	0.798	0.694–0.902	0.553	0.941	0.913	0.653	0.913	0.553	0.689	0.564	Nomogram

Abbreviations: AUC, the area under the curve; CI, confidence interval; NPV, negative predictive value; PPV, positive predictive value.

This study successfully leveraged the synergy of radiomics and machine learning to create an innovative model for predicting the efficacy of PD‐1 inhibitors in treating extensive‐stage SCLC. The model showcased high accuracy and exemplary performance, reliably forecasting the response of SCLC patients to PD‐1 inhibitor therapy. These findings underscore the model's potential as a valuable, non‐invasive tool in guiding clinical decision‐making and optimizing treatment strategies for SCLC. By offering insights into patient‐specific responses to PD‐1 inhibitors, this model represents a significant step forward in personalized cancer care, potentially improving treatment outcomes for individuals with extensive‐stage SCLC.

## CONFLICT OF INTEREST STATEMENT

The authors declare no conflict of interest.

## FUNDING INFORMATION

Young Jianghuai famous medical training project, Excellent physician Training program of Anhui Medical University, Research Fund project of Anhui Medical University (2023xkj144) and Research Fund of Anhui Institute of translational medicine (2023zhyx‐C40).

## ETHICS STATEMENT

The study protocol underwent review and approval by the Institutional Review Committee of the First Affiliated Hospital of Anhui Medical University (Quick ‐PJ 2023‐12‐53), The First Affiliated Hospital of Soochow University ((2023) Batch No:371) and Dushu Lake Hospital of Soochow University (2023. Batch No:230133). The study was a multicenter retrospective study, and the Ethics Committee agreed to waive informed consent.

## Supporting information

Supporting Information

## Data Availability

The original contributions presented in the study are included in the article and the Supporting Information.
