# Supplementary material for: Machine learning and radiomics for predicting efficacy of programmed cell death protein 1 inhibitor for small cell lung cancer: A multicenter cohort study
Source: Clin Transl Med. 2024 Jun 5;14(6):e1673. doi: 10.1002/ctm2.1673 (PMC11154803; doi:10.1002/ctm2.1673)
Supplement: Supplementary file 1 — Supporting Information [file CTM2-14-e1673-s001.docx]

| Variables | Training cohort(n=129) | | Internal validation cohort(n=32) | | External validation cohort(n=72) | | All cohort(n=233) | | p valve |
| --- | --- | --- | --- | --- | --- | --- | --- | --- | --- |
|  | Non- Responses (n=69) | Responses (n=60) | Non- Responses (n=17) | Responses (n=15) | Non- Responses (n=38) | Responses (n=34) | Non- Responses (n=124) | Responses (n=109) |  |
| Age(years) | 65.88(8.13) | 64.08(9.23) | 67.06(7.96) | 65.40(9.36) | 63.97(8.25) | 63.85(9.77) | 65.46(8.14) | 64.19(9.34) | 0.234 |
| CEA(ng/ml) | 16.83(50.66) | 9.17(25.48) | 65.50(206.59) | 6.78(14.57) | 26.58(71.72) | 26.36(63.01) | 26.49(93.70) | 14.21(40.78) | 0.839 |
| NSE(ng/ml) | 69.44(88.53) | 44.65(59.17) | 75.44(99.65) | 77.71(97.08) | 81.77(101.90) | 33.21(50.27) | 74.03(93.69) | 45.62(64.02) | 0.018 |
| CY211(ng/ml) | 6.36(8.01) | 3.87(2.88) | 5.67(6.64) | 3.77(2.81) | 8.19(24.92) | 10.81(31.75) | 6.83(15.13) | 6.02(18.00) | 0.255 |
| NLR | 3.68(3.32) | 3.96(3.57) | 5.09(3.90) | 2.95(1.88) | 3.56(2.27) | 4.37(5.12) | 3.84(3.14) | 3.95(3.95) | 0.302 |
| PLR | 159.99(80.44) | 178.88(91.88) | 170.65(119.35) | 160.67(73.67) | 170.68(83.59) | 192.14(111.65) | 164.73(86.95) | 180.51(96.04) | 0.185 |
| Tumer diameter(cm) | 3.83(2.05) | 3.74(1.91) | 3.89(1.77) | 3.66(1.92) | 4.35(2.04) | 3.94(1.83) | 4(2.01) | 3.79(1.87) | 0.796 |
| Sex |  |  |  |  |  |  |  |  | 0.03 |
| Female | 11(15.94) | 13(21.67) | 2(11.76) | 3(20.00) | 3(7.89) | 10(29.41) | 16(12.90) | 26(23.85) |  |
| Male | 58(84.06) | 47(78.33) | 15(88.24) | 12(80.00) | 35(92.11) | 24(70.59) | 108(87.10) | 83(76.15) |  |
| Smoking history |  |  |  |  |  |  |  |  | 0.546 |
| Nonsmoker | 32(46.38) | 29(48.33) | 7(41.18) | 3(20.00) | 13(34.21) | 18(52.94) | 52(41.94) | 50(45.87) |  |
| Smoker | 37(53.62) | 31(51.67) | 10(58.82) | 12(80.00) | 25(65.79) | 16(47.06) | 72(58.06) | 59(54.13) |  |
| ECOG PS score |  |  |  |  |  |  |  |  | 0.256 |
| 0 | 11(15.94) | 7(11.67) | 3(17.65) | 1(6.67) | 4(10.53) | 1(2.94) | 18(14.51) | 9(8.26) |  |
| 1 | 56(81.16) | 49(81.67) | 14(82.35) | 13(86.67) | 32(84.21) | 31(91.18) | 102(82.26) | 93(85.32) |  |
| 2 | 2(2.90) | 3(5.00) | 0 | 0 | 2(5.26) | 2(5.88) | 4(3.23) | 5(3.86) |  |
| 3 | 0 | 1(1.67) | 0 | 1(6.67) | 0 | 0 | 0 |  |  |

**Table S1. Baseline data for patients with SCLC in the training and validation cohorts.**

Continuous variable data are presented as the mean (SD) or the median (IQR). Classified variable data are presented as n (%). *p* values are based on comparisons between the Non-Responses and Responses group in All cohort. CY211, Cytokeratin 19 fragment, CEA, carcinoma embryonic antigen, NSE, neuron specific enolase, NLR, Neutrophil to Lymphocyte ratio, PLR, platelet to lymphocyte ratio.

**Table S2.** Univariate and multivariate analysis of clinical variables.

|  | Univariate analysis |  | Multivariate analysis |  |
| --- | --- | --- | --- | --- |
| Characteristics | OR(95% CI) | P value | OR(95% CI) | P value |
| age | 0.983 (0.955 - 1.013) | 0.269 |  |  |
| sex |  |  |  |  |
| female | Reference |  | Reference |  |
| male | 0.473 (0.238 - 0.939) | 0.032 | 0.511 (0.256 - 1.022) | 0.058 |
| smoker |  |  |  |  |
| yes | Reference |  |  |  |
| no | 0.852 (0.507 - 1.432) | 0.546 |  |  |
| ECOGPS |  |  |  |  |
| 1 | Reference |  |  |  |
| 2 | 1.371 (0.357 - 5.259) | 0.646 |  |  |
| 0 | 0.548 (0.235 - 1.281) | 0.165 |  |  |
| CEA | 0.997 (0.992 - 1.002) | 0.238 |  |  |
| NSE | 0.995 (0.991 - 0.999) | 0.012 | 0.995 (0.992 - 0.999) | 0.019 |
| CY211 | 0.997 (0.981 - 1.013) | 0.711 |  |  |
| NLR | 1.009 (0.938 - 1.086) | 0.803 |  |  |
| PLR | 1.002 (0.999 - 1.005) | 0.191 |  |  |
| tumer_diameter | 0.946 (0.828 - 1.082) | 0.419 |  |  |

CY211, Cytokeratin 19 fragment, CEA, carcinoma embryonic antigen, NSE, neuron specific enolase, NLR, Neutrophil to Lymphocyte ratio, PLR, platelet to lymphocyte ratio.

| Feature Selected | Group | coefficient |
| --- | --- | --- |
| wavelet_HHL_firstorder_Median | firstorder | 0.045979 |
| original_shape_Flatness | shape | -0.011089 |
| log_sigma_1_0_mm_3D_glcm_Correlation | glcm | -0.02064 |
| lbp_3D_k_glszm_GrayLevelNonUniformityNormalized | glszm | -0.020886 |
| lbp_3D_m2_glcm_ClusterShade | glcm | -0.114084 |

**Table S3.** The selected radiomics features.

**Figure S1**. The flow diagram of the study.


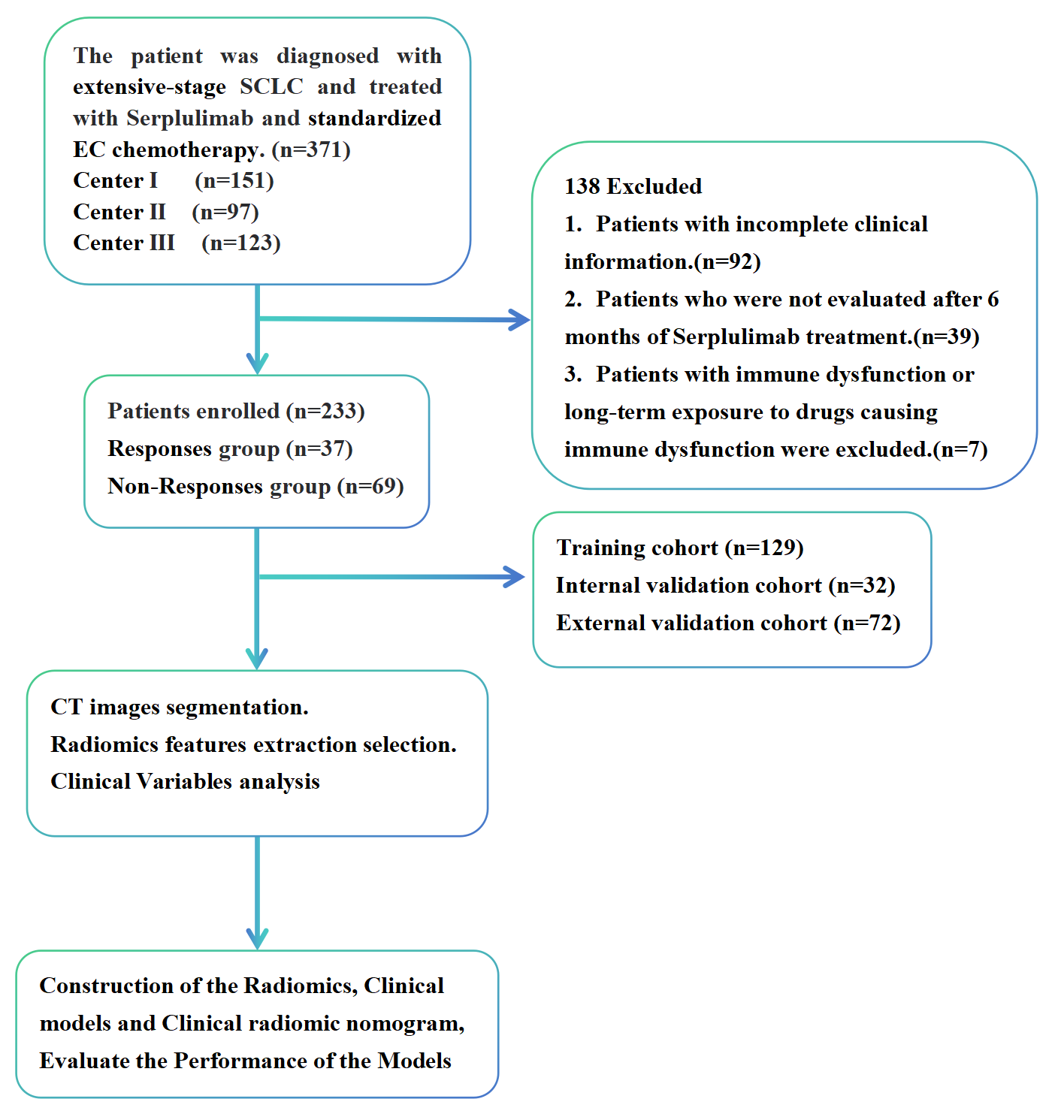


*Note: The chemotherapy regimen was the EC regimen: etoposide (100 mg/m2, days 1-3) combined with carboplatin (AUC 5-6, day 1), repeated every 3 weeks for 4-6 cycles. The Serplulimab dose was set at 4.5mg/kg every 3 weeks until disease progression or intolerable toxicity occurred.

**Figure S2**. Univariate and multivariate analysis of clinical variables shown in forest map.


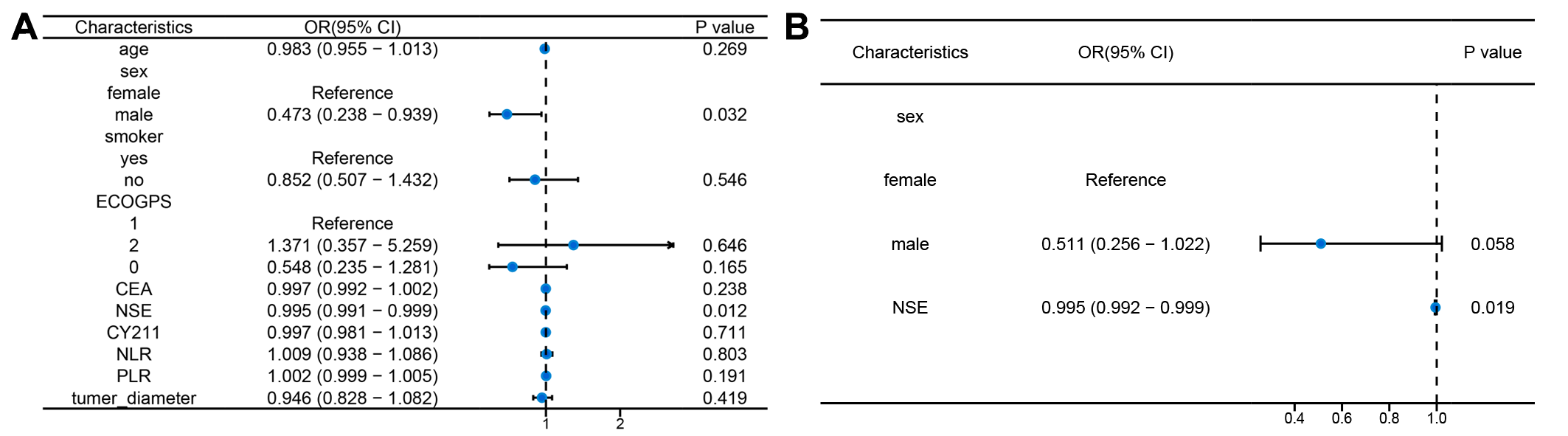


**Figure S3.** Radiomic feature selection results. A). The histogram represents the coefficients of the selected radiomic features. B). The weights of the selected radiomic features in LGBMClassifier.


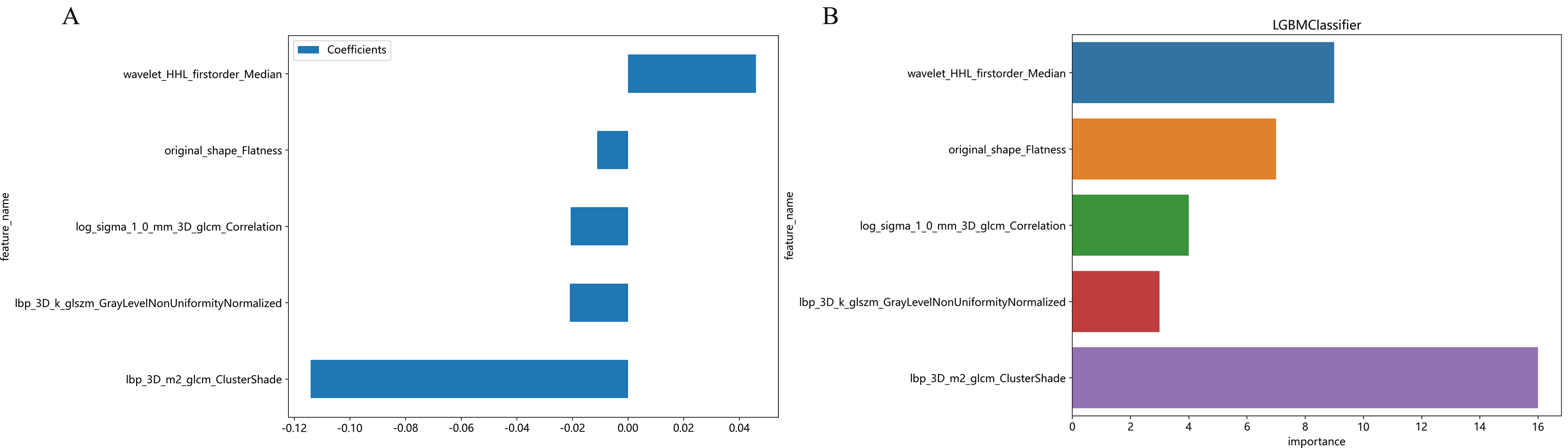


**Appendix**

Appendix 1．Criteria for evaluating the effectiveness of immunotherapy:

The effectiveness of immunotherapy was measured based on durable benefit (DCB) or no durable benefit (NDB)[1]. Response Evaluation Criteria in Solid Tumors (RECIST1.1) was used to define progression[2]. The Response Evaluation Criteria in Solid Tumors (RECIST1.1) defined progression[3]. The objective response rate (ORR) was used to assess response to PD-1 inhibitors. The response group included patients achieving complete response (CR) or partial response (PR), while the no response group comprised patients with stable disease (SD) or progression disease (PD). Due to the length of the letter, we could not add this part to the body of the letter, so we added the explanation of this part to the appendix of the supplementary document.

[1]. Rizvi NA, Hellmann MD, Snyder A, Kvistborg P, Makarov V, Havel JJ, Lee W, Yuan J, Wong P, Ho TS *et al*: **Cancer immunology. Mutational landscape determines sensitivity to PD-1 blockade in non-small cell lung cancer**. *Science (New York, NY)* 2015, **348**(6230):124-128.

[2]. Eisenhauer EA, Therasse P, Bogaerts J, Schwartz LH, Sargent D, Ford R, Dancey J, Arbuck S, Gwyther S, Mooney M *et al*: **New response evaluation criteria in solid tumours: revised RECIST guideline (version 1.1)**. *European journal of cancer (Oxford, England : 1990)* 2009, **45**(2):228-247.

[3]. Cheng Y, Han L, Wu L, Chen J, Sun H, Wen G, Ji Y, Dvorkin M, Shi J, Pan Z *et al*: **Effect of First-Line Serplulimab vs Placebo Added to Chemotherapy on Survival in Patients With Extensive-Stage Small Cell Lung Cancer: The ASTRUM-005 Randomized Clinical Trial**. *Jama* 2022, **328**(12):1223-1232.

**Appendix 2．**

**Radiomics feature screening process:**

This study employed rigorous data preprocessing and feature selection methodologies to ensure the reliability and validity of the radiomic features used. Interobserver reliability and intraobserver repeatability, crucial for the robustness of radiomic feature extraction, were assessed using inter-class and intra-class correlation coefficients (ICCs). Features exhibiting an ICC value below 0.75 were eliminated from our analysis to maintain a high standard of data consistency and reliability. Subsequently, a T-test was conducted to identify features with significant variance between groups, with a threshold p-value set at less than 0.05. This step was critical in narrowing down the pool of features to those most likely to yield meaningful insights in univariate analysis. The final stage of feature selection involved the application of least absolute shrinkage and selection operator (LASSO) regression analysis. This powerful statistical technique was used to pinpoint statistically significant radiomic features that effectively differentiate between the treatment response and non-response groups within our patient cohort. To mitigate the risk of overfitting—a common pitfall in model development—a five-fold cross-validation approach was implemented. This strategy enhances the generalizability and validity of the LASSO regression analysis, ensuring that the selected features are robust and reliable predictors across different subsets of the data.

**Clinical features screening process:**

Univariate and multivariate logistic regression analyses were conducted to identify clinical variables showing significant differences between the response and non-response groups (P < 0.05). These identified variables were then used to construct the clinical models.
